# The protective role of daidzein in intestinal health of turbot (*Scophthalmus maximus* L.) fed soybean meal-based diets

**DOI:** 10.1038/s41598-021-82866-1

**Published:** 2021-02-08

**Authors:** Guijuan Yu, Yang Liu, Weihao Ou, Jihong Dai, Qinghui Ai, Wenbing Zhang, Kangsen Mai, Yanjiao Zhang

**Affiliations:** 1 The Key Laboratory of Aquaculture Nutrition and Feed (Ministry of Agriculture), and the Key Laboratory of Mariculture (Ministry of Education), Ocean University of China, Qingdao, China; 2 Laboratory for Marine Fisheries Science and Food Production Processes, Qingdao National Laboratory for Marine Science and Technology, Qingdao, China

**Keywords:** Zoology, Animal physiology

## Abstract

Soybean meal-induced enteropathy (SBMIE) is prevalent in aquaculture. The aim of this study is to evaluate the role of daidzein on SBMIE of juvenile turbot (*Scophthalmus maximus* L.) by feeding with fish meal diet (FM), soybean meal diet (SBM, 40% fish meal protein in FM replaced by soybean meal protein) and daidzein diet (DAID, 40 mg/kg daidzein supplemented to SBM) for 12 weeks. We found that daidzein supplementation elevated the gene expression of anti-inflammatory cytokine *TGF-β*, decreased gene expression of pro-inflammatory cytokines *TNF-α* and signal molecules *p38*, *JNK* and *NF-κB*. SBM up-regulated the genes expression related to oxidative stress and apoptosis, but dietary daidzein restored it to the similar level with that in FM group. Moreover, dietary daidzein up-regulated gene expression of tight junction protein, and modified the intestinal microbial profiles with boosted relative abundance of phylum Proteobacteria and Deinococcus–Thermus, genera *Sphingomonas* and *Thermus*, species *Lactococcus lactis*, and decreased abundance of some potential pathogenic bacteria. In conclusion, dietary daidzein could ameliorate SBM-induced intestinal inflammatory response, oxidative stress, mucosal barrier injury and microbiota community disorder of turbot. Moreover, p38, JNK and NF-κB signaling might be involved in the anti-inflammatory process of daidzein, and daidzein itself might act as an antioxidant to resist SBM-induced oxidative damage.

## Introduction

Intestine, the important interface between the host and the external environment, is continually exposed to the external stimulations such as diets and environmental factors, which makes it vulnerable to damage^1^. Due to the mass use of soybean meal in aquafeed, soybean meal-induced enteropathy (SBMIE) prevailed in fish especially in marine fish like Atlantic salmon (*Salmo salar* L.)^2^, turbot (*Scophthalmus maximus* L.)^3,4^, rainbow trout (*Oncorhynchus mykiss*)^5^, European sea bass (*Dicentrarchus labrax*)^6^ and red drum (*Sciaenops ocellatus*)^7^. SBMIE is mainly characterized by release of inflammatory cytokines^8,9^, oxidative damage^10^, apoptosis of intestinal cell^11,12^, disruption of tight junction^13,14^, as well as intestinal microbiota disorders^15,16^, which seriously affected the health and growth performance of fish. Thus, an effective and environmentally approach to prevent the enteropathy is imperative, and would be beneficial to the use of soybean meal in aquafeed.

Recently, nutritional strategies blossom quickly in aquaculture, especially in functional additives. Daidzein, one of the most abundant isoflavones extracted from soy, is a hormone-like substance with many biological activities^17–19^. Numerous studies on mammals and human cancer cell show that daidzein could attenuate inflammation and oxidative stress, induce the apoptosis of cancer cell, and modulate the intestinal bacterial composition^18,20,21^. As a natural anti-inflammatory agent, daidzein has been reported to be able to ameliorate dextran sulfate sodium (DSS)-induced colitis in mouse by inhibiting nuclear transcription factor-κB (NF-κB) signaling^22^. The Keap1-Nuclear factor E2-related factor 2 (Nrf2) pathway is the major defense mechanism to counteract oxidative stress^23^. A previous study demonstrated that isoflavones (composed of 55% genistein, 23% daidzein, and 14% glycitein) therapy improved antioxidant capacities in ischemic cardiomyopathy patients by activating Nrf2-mediated antioxidant responses^24^. Recently, Tomar et al.^21^ found that mitogen-activated protein kinase (MAPK) signaling was crucial in daidzein-mitigated inflammation, oxidative stress and apoptosis in cisplatin-induced kidney injury of rats. In fish, relevant research is limited. In our previous studies, the possibility of daidzein as a functional additive in fish was assessed^25–27^, and the beneficial effects of dietary daidzein were observed. However, the roles of daidzein in mitigating adverse effects of high soybean meal level on the intestinal health, as well as the mechanisms involved, remain unknown.

Turbot is an extensively cultured marine carnivorous fish in the world. The purpose of the present study was to explore the effects of daidzein on SBMIE of turbot in terms of the intestinal inflammation, oxidative stress, intestinal integrity and microbiota, as well as the mechanisms involved, which would provide a better understanding of daidzein physiology in fish.

## Results

### Gene expression of cytokines and signaling molecules

**Figure 1:**
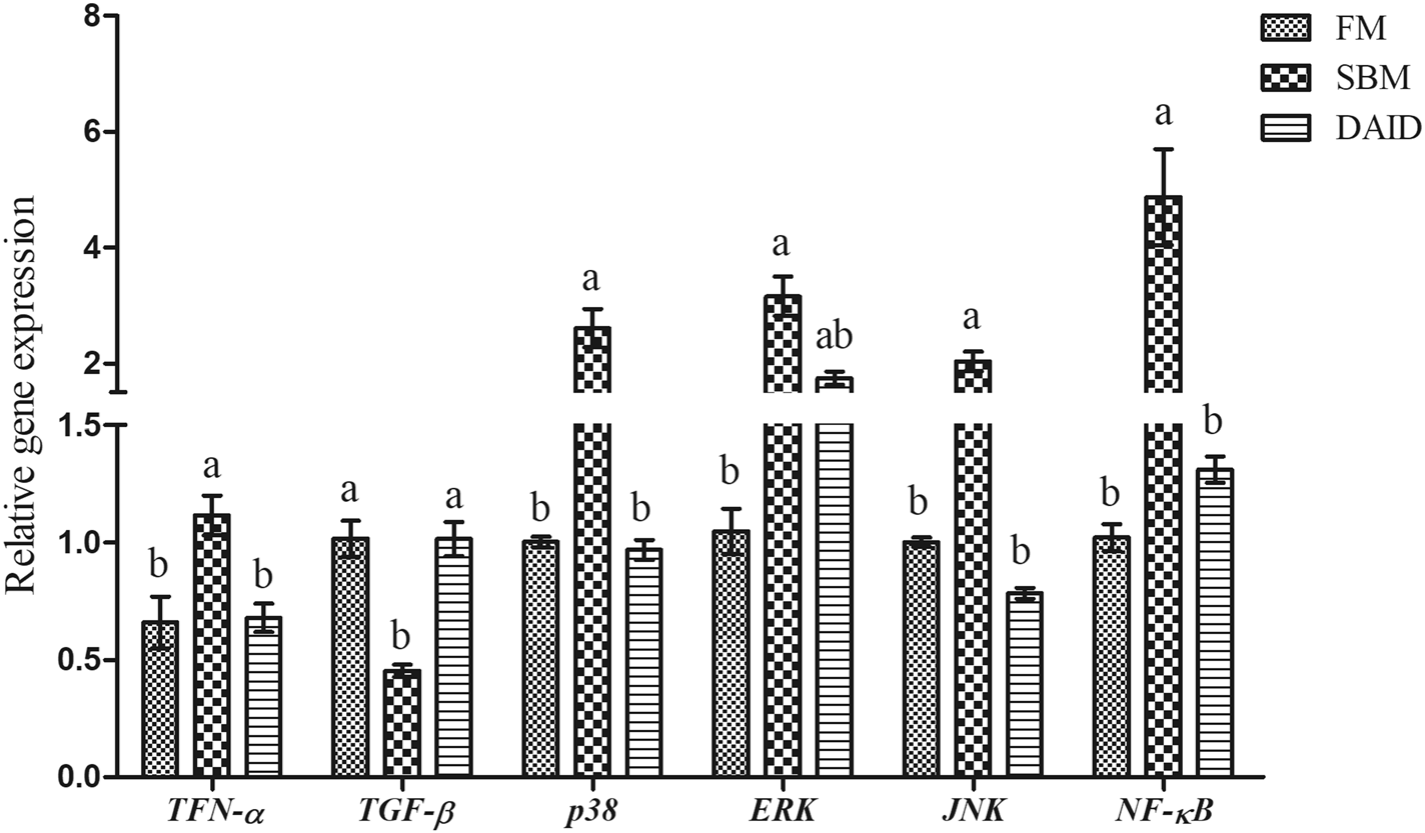
Effects of daidzein on gene expression of cytokines and signaling molecules of turbot fed with soybean meal. *FM* fish meal diet, *SBM* soybean meal diet, *DAID* 40 mg/kg daidzein included into SBM diet, *TNF-α* tumor necrosis factor-α, *p38* p38 mitogen-activated protein kinase, *TGF-β* transforming growth factor-β, *JNK* c-Jun N-terminal kinase, *ERK* extracellular regulated kinase; *NF-κB* nuclear transcription factor-kappa B; Values are mean ± SEM, n = 3 and values shared different letters are significantly different (*P* < 0.05).

Diet SBM significantly up-regulated the mRNA expression of pro-inflammatory cytokine tumor necrosis factor-α (*TNF-α*) and signaling molecules *p38*, extracellular regulated kinase (*ERK*), c-Jun N-terminal kinase (*JNK*) and *NF-κB*, and down-regulated the expression of anti-inflammatory cytokine transforming growth factor-β (*TGF-β*) (*P* < 0.05) (Fig. 1). However, the daidzein supplementation significantly suppressed the expression of *TNF-α*, *p38*, *JNK* and *NF-κB*, and increased *TGF-β* expression compared with SBM group (*P* < 0.05).

### Gene expression related to oxidative stress

**Figure 2:**
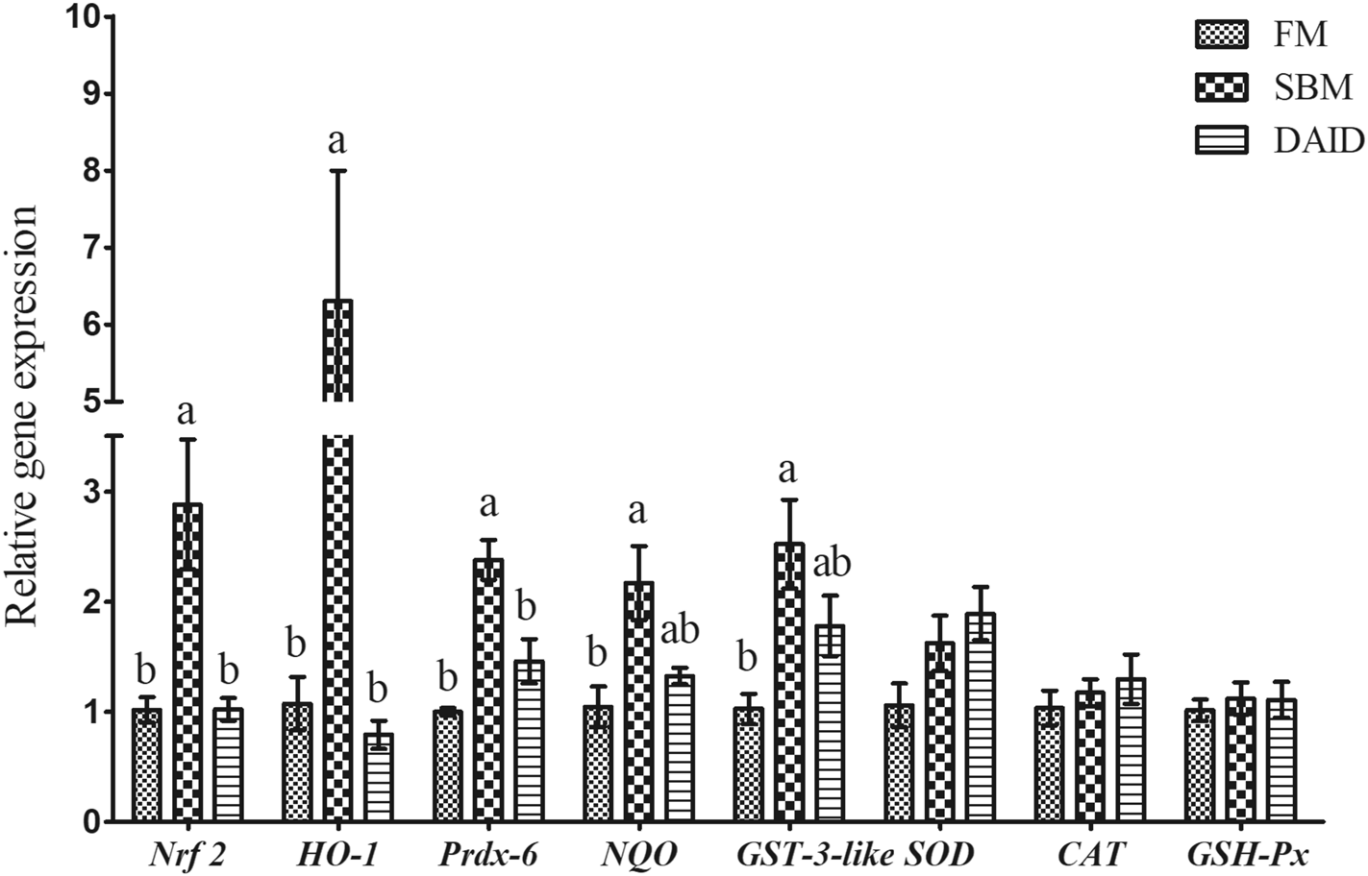
Effects of daidzein on gene expression related to oxidative stress of turbot fed with soybean meal. *FM* fish meal diet, *SBM* soybean meal diet, *DAID* 40 mg/kg daidzein included into SBM diet, *Nrf2* nuclear factor E2-related factor 2, *HO-1* heme oxygenase-1, *Prdx-6* Peroxiredoxin-6, *NQO* NAD(P)H quinone oxidoreductase, *GST-3-like* glutathione-*S*-transferase-3-like, *SOD* superoxide dismutase, *CAT* catalase, *GSH-Px* glutathione peroxidase. Values are mean ± SEM, n = 3 and values shared different letters are significantly different (*P* < 0.05).

Diet SBM resulted in significantly up-regulated gene expression of *Nrf2*, Heme oxygenase-1(*HO-1*), Peroxiredoxin-6 (*Prdx-6*), NAD (P)H quinone dehydrogenase (*NQO*) and Glutathione-*S*-transferase-3-like (*GST-3-like*) compared to fish meal (FM) group (*P* < 0.05), while daidzein supplementation suppressed the gene expression of *Nrf2*, *HO-1*, *Prdx-6*, *NQO* and *GST-3-like* to the similar level of the FM group (Fig. 2). There was no significant difference of *SOD*, *CAT* and *GSH-Px* among treatments.

### Gene expression of apoptotic parameters

**Figure 3:**
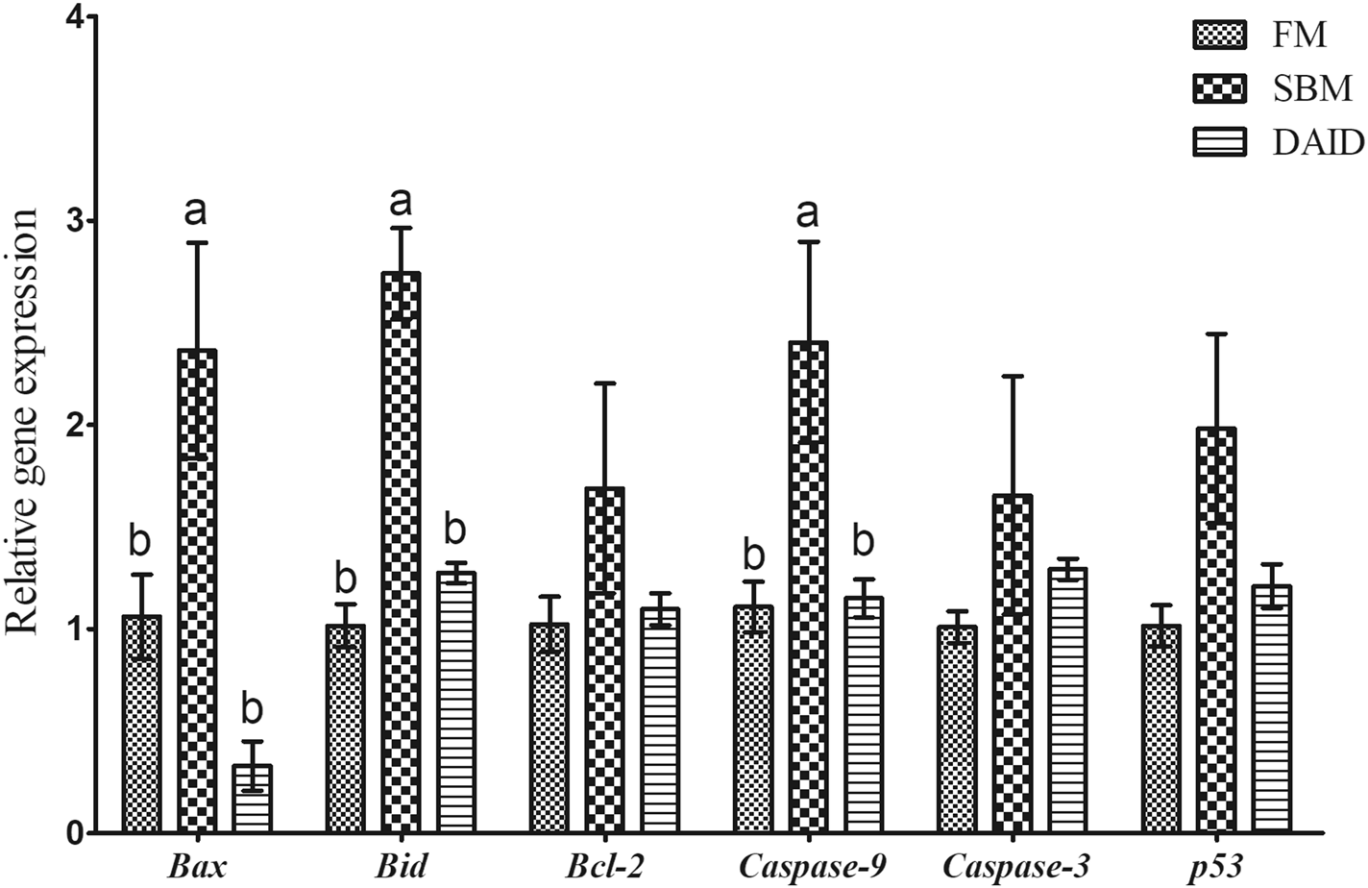
Effects of daidzein on gene expression of apoptotic parameters of turbot fed with soybean meal. *FM* fish meal diet, *SBM* soybean meal diet, *DAID* 40 mg/kg daidzein included into SBM diet. Values are mean ± SEM, n = 3 and values shared different letters are significantly different (*P* < 0.05).

Diet SBM significantly elevated the mRNA levels of *Bax*, *Bid* and *caspase-9*, and these parameters were suppressed by 40 mg/kg daidzein (*P* < 0.05) (Fig. 3). Besides, no significant difference was observed in the mRNA levels of *Bcl-2*, *caspase-3* and *p53* (*P* > 0.05) among all groups.

### Gene expression of tight junctions (TJs) proteins

**Figure 4:**
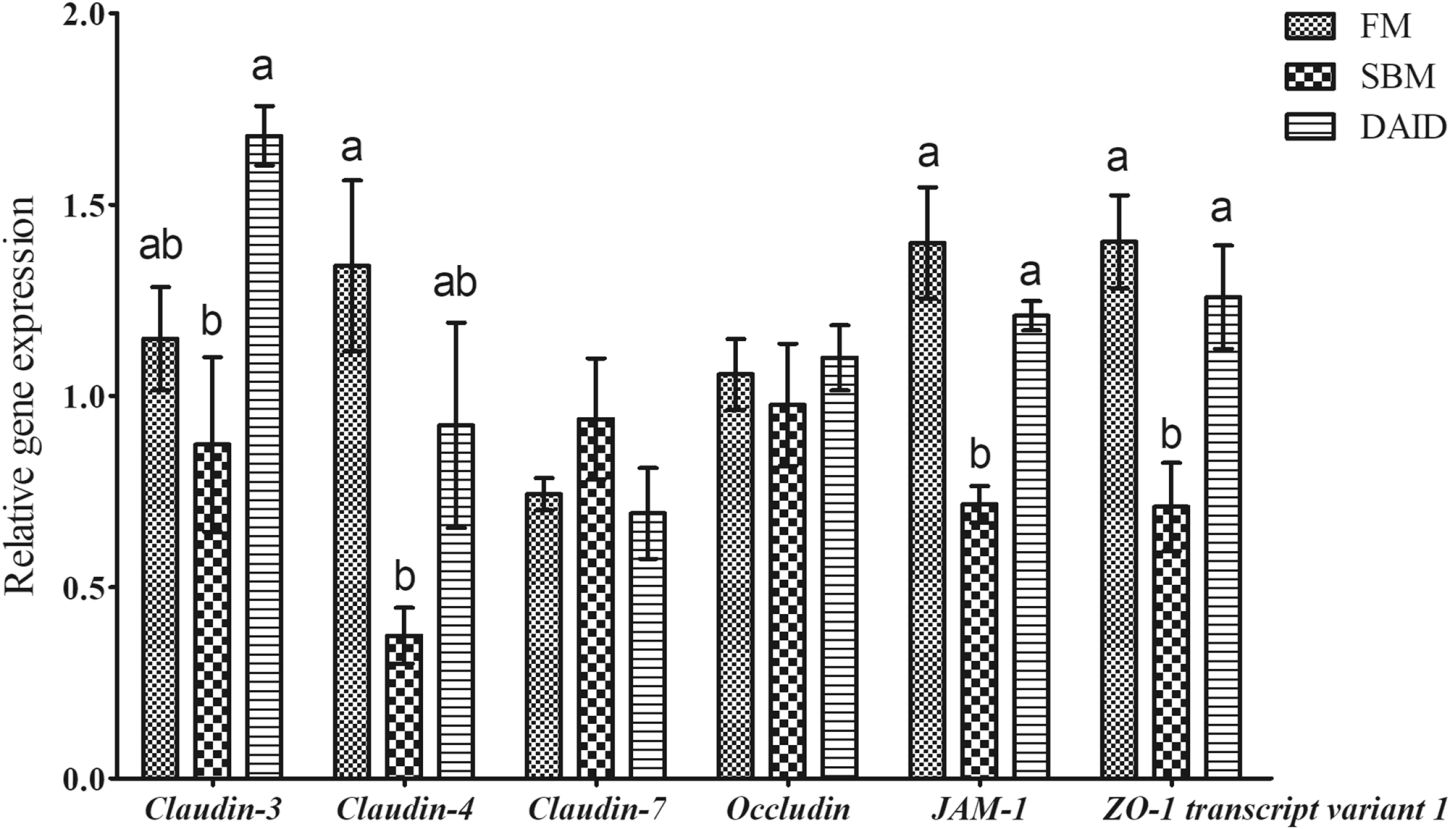
Effects of daidzein on gene expression of tight junction proteins of turbot fed with soybean meal. *FM* fish meal diet, *SBM* soybean meal diet, *DAID* 40 mg/kg daidzein included into SBM diet, *JAM-1* Junctional adhesion molecules-1, *ZO-1* Zonula occludens-1. Values are mean ± SEM, n = 3 and values shared different letters are significantly different (*P* < 0.05).

Compared with the FM group, diet SBM significantly reduced the gene expression of *claudin-4*, Junctional adhesion molecules-1 (*JAM-1*), and Zonula occludens-1 (*ZO-1*) *transcript variant 1* (*P* < 0.05), while dietary 40 mg/kg daidzein reversed these changes showing significantly up-regulated gene expression of *claudin-3*, *JAM-1*, and *ZO-1 transcript variant 1* (*P* < 0.05) and increased gene expression of *claudin-4* (*P* > 0.05) (Fig. 4).

### Intestinal microbiota

**Figure 5:**
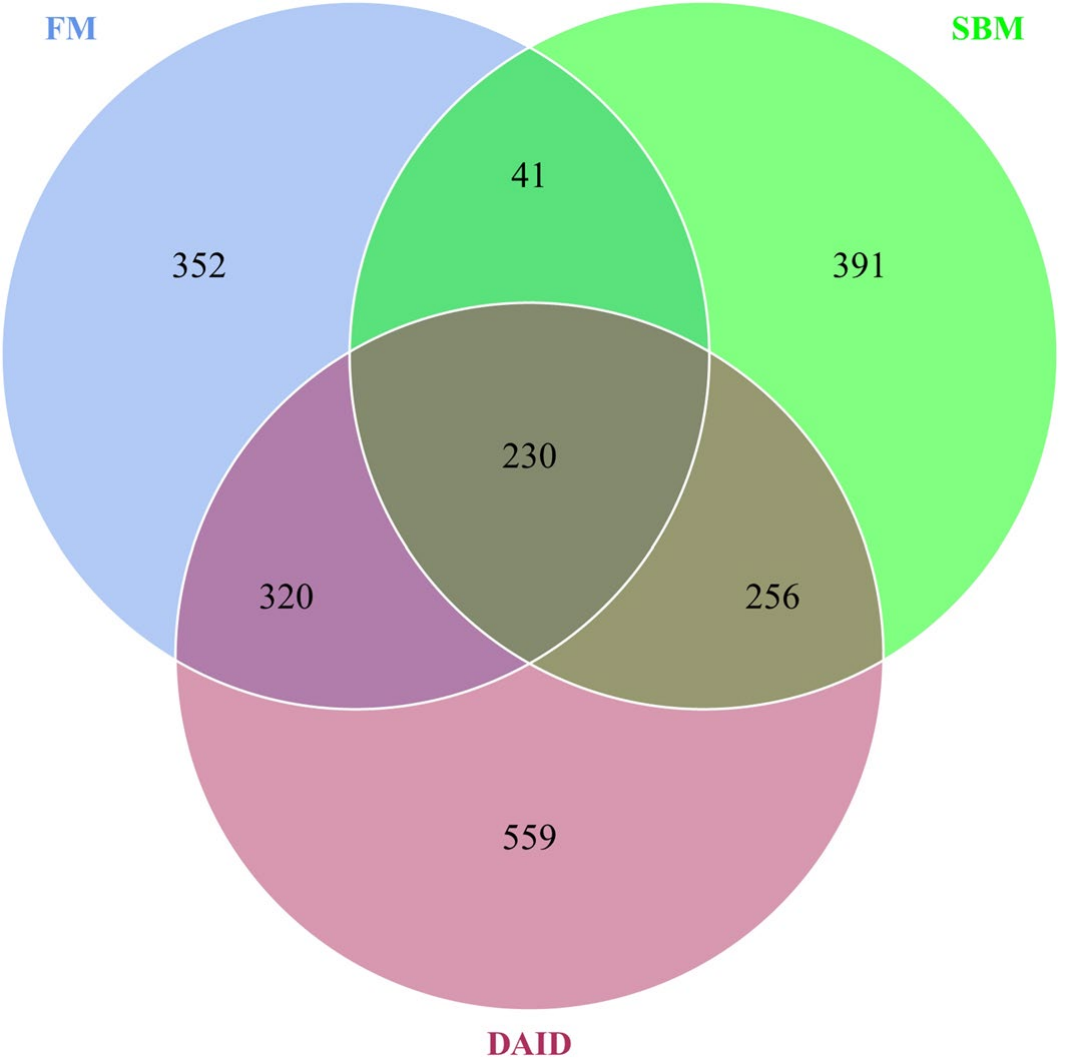
Venn diagram of intestinal microbiota. *FM* fish meal diet, *SBM* soybean meal diet, *DAID* 40 mg/kg daidzein included into SBM diet.

The observed species number of all samples reached the saturation phase, suggesting adequate sequencing depth (Supplementary Fig. S1). A Venn diagram showed that FM and SBM shared 271 operational taxonomic units (OTUs), while, FM and DAID (daidzein group) shared 550 OTUs (Fig. 5).

**Figure 6:**
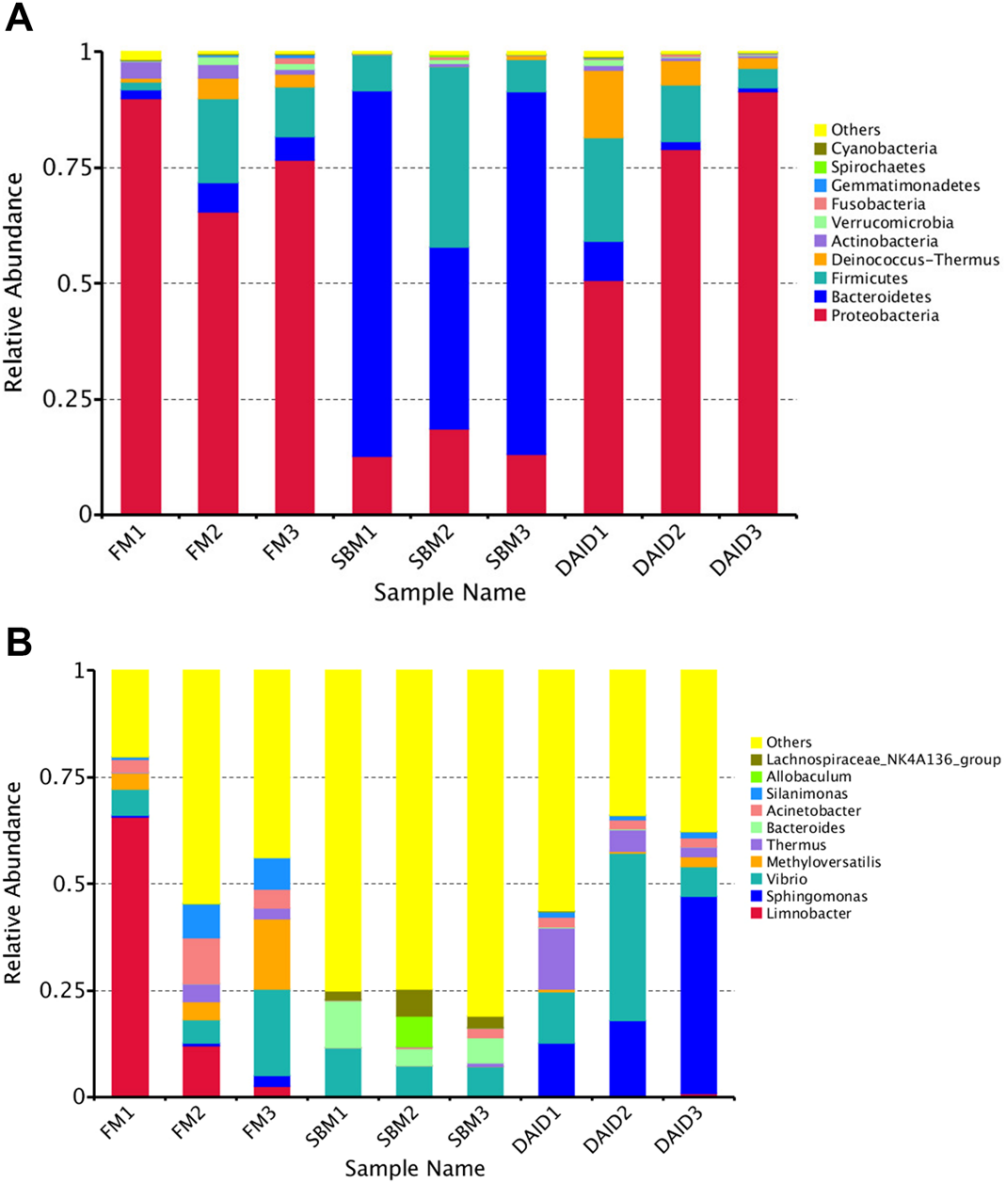
Top 10 most abundant (based on relative abundance) bacterial phyla (**A**) and genera (**B**). *FM* fish meal diet, *SBM* soybean meal diet, *DAID* 40 mg/kg daidzein included into SBM diet.

**Figure 7:**
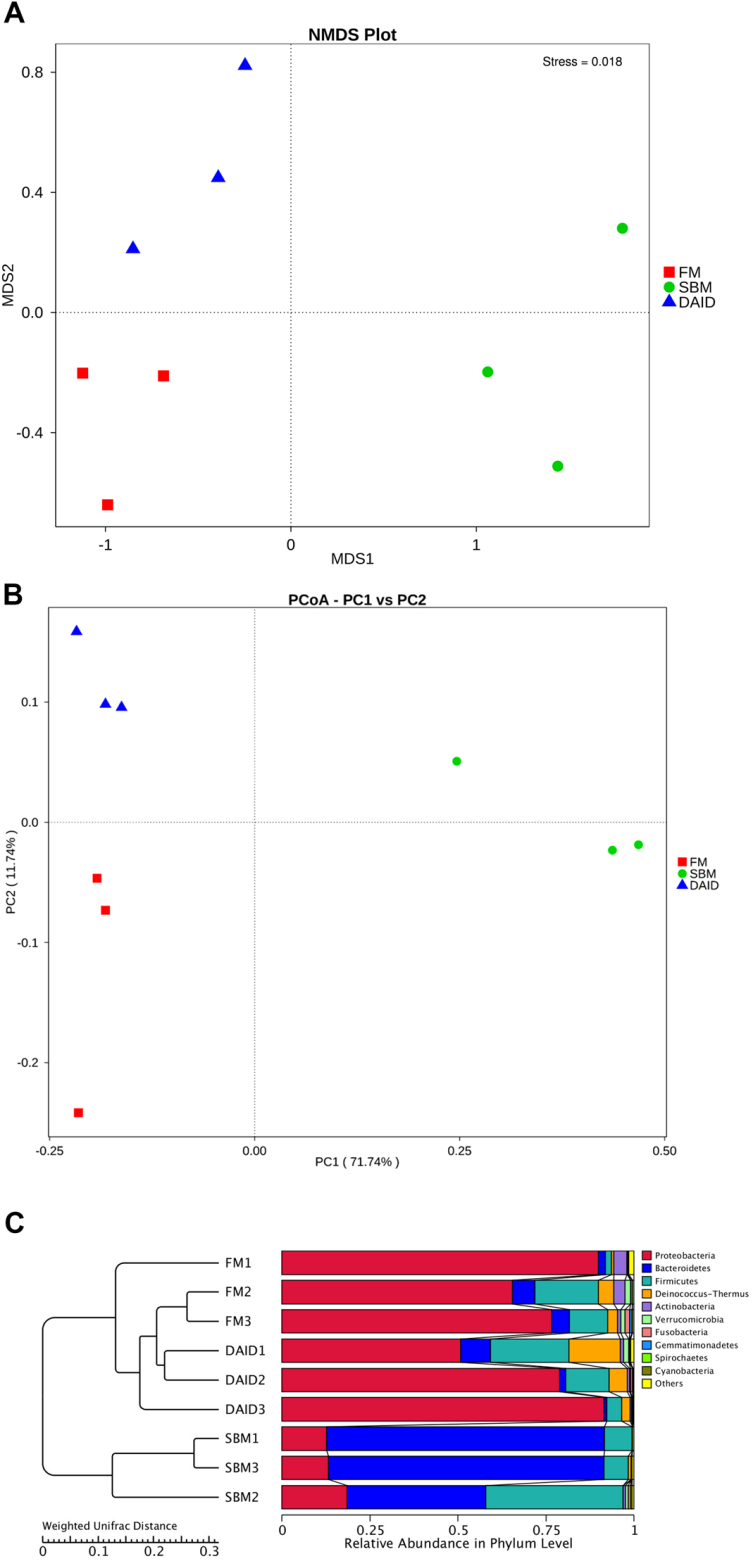
Beta diversity of intestinal microbiota of juvenile turbot. (**A**) non-metric multi-dimensional scaling (NMDS) analysis; (**B**) principal coordinate analysis (PCoA) based on weighted unifrac distances; (**C**) unweighted pair-group method with arithmetic mean (UPGMA)-clustering trees based on weighted unifrac distances. *FM* fish meal diet, *SBM* soybean meal diet, *DAID* 40 mg/kg daidzein included into SBM diet.

**Figure 8:**
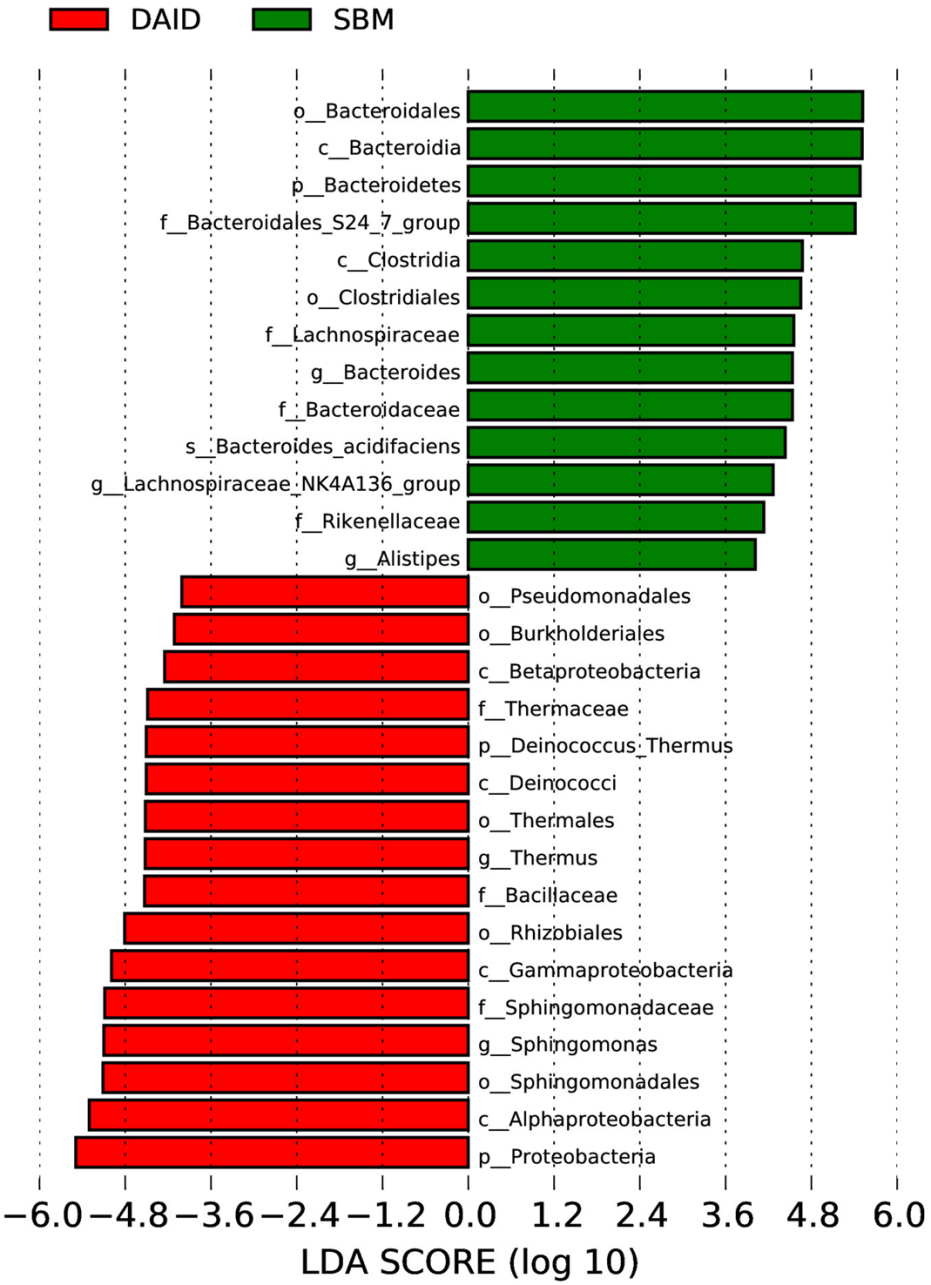
Linear discriminant analysis (LDA) score distribution bar graph of LEfSe analysis on intestinal microbiota of juvenile turbot. The LDA score greater than 4 was considered differentiated species. The letter (p, c, o, f, g and s) in front of phylotype names represented phylum, class, order, family, genus and species level respectively. The different colors of the bars indicated corresponding groups (red: DAID, green: SBM). *SBM* soybean meal diet, *DAID* 40 mg/kg daidzein included into SBM diet.

At phylum and genus levels, the top four predominant bacterial phyla were Proteobacteria, Bacteroidetes, Firmicutes, and Deinococcus–Thermus (Fig. 6A), the top four dominant genera were *Limnobacter*, *Sphingomonas*, *Vibrio* and *Methyloversatilis* in distal intestine of turbot (Fig. 6B). The results of α-diversity did not show any difference among all groups (Supplementary Table S1). The β-diversity analysis, such as Non-Metric Multi-Dimensional Scaling (NMDS) (Fig. 7A), principal co-ordinates analysis (PCoA) (Fig. 7B) as well as Unweighted Pair-group Method with Arithmetic Mean (UPGMA) analysis (Fig. 7C) based on weighted unifrac distances, indicates that the clusters and intestinal bacteria composition of DAID were more similar to FM group, and distinctly separated from SBM. MetaStat analysis on genus and species level showed that daidzein significantly boosted the abundance of *unidentified_Desulfobacteraceae*, *unidentified_Xanthomonadales*, *bacterium_enrichment_culture_clone_AOM-SR-B34*, *Steroidobacter_sp._*WWH78 and *Lactococcus lactis* (Table 1). Besides, according to analysis of linear discriminant analysis (LDA) Effect Size (LEfSe), at phylum level, the supplementation of daidzein strikingly increased the abundance of Proteobacteria and Deinococcus–Thermus, and decreased the abundance of Bacteroidetes (Fig. 8). Moreover, daidzein highly decreased the relative abundance of family *Bacteroidales S24-7* from 53.00% (SBM group) to 0.25% (DAID group) (Supplementary Table S2). At genus and species levels, daidzein significantly increased the relative abundance of *Sphingomonas* and *Thermus*, and significantly decreased the relative abundance of *Alistipes*, *Lachnospiraceae* NK4A136, *Bacteroides* and *Bacteroides acidifaciens* compared with the SBM group (Fig. 8).

**Table 1:** The MetaStat analysis of intestinal microbiota of turbot at genus and species levels (× 10^−5^).

	SBM		DAID	
	Mean	SEM	Mean	SEM
**Genus**				
*Unidentified_Desulfobacteraceae*	0^b^	0	4.43^a^	0
*Unidentified_Xanthomonadales*	0^b^	0	4.43^a^	0
**Species**				
*Steroidobacter_sp._WWH78*	0^b^	0	4.43^a^	0
*Lactococcus lactis*	0^b^	0	4.43^a^	0
*bacterium_enrichment_culture_clone_AOM-SR-B34*	0^b^	0	4.43^a^	0

Values shared different letters in the same row are significantly different (q-value (corrected *p* value) < 0.05), n = 3.

*FM* fish meal diet, *SBM* soybean meal diet, *DAID* 40 mg/kg daidzein included into SBM diet.

## Discussion

Our previous study showed that dietary daidzein improved SBM induced typical inflammatory symptoms such as profound infiltration of eosinophil, disarranged microvilli, wizened and shorter intestinal folds^28^. However, the involved mechanisms were still unclear. The release of inflammatory cytokines plays an important role in mediating SBMIE. In present study, dietary daidzein significantly down-regulated the gene expression of pro-inflammatory cytokine *TNF-α* and up-regulated the expression of anti-inflammatory cytokine *TGF-β*. These results were consistent with the study on DSS-induced colitis mouse model, which showed oral administration of daidzein ameliorated the ulcerative colitis and suppressed the expression of TNF-α, interleukin-1β (IL-1β), and IL-6^22^. The decreased gene expression of pro-inflammatory cytokines responded to daidzein was also found in intestine of turbot fed with fish meal-based diet^27^. It has been reported that many signaling pathways are involved in the anti-inflammatory effects of daidzein, of which MAPK and NF-κB are the most deeply studied^21,22,29^. MAPKs, a family of serine/threonine protein kinases, are potential molecular targets for anti-inflammatory therapeutics^30^, the classical MAPKs are consisted of ERK1/2, p38, JNK and ERK5^31^. NF-κB is a central mediator involved in inflammation development and progression^32^. Tomar et al.^21^ found pretreatment with 100 mg/kg daidzein diminished the activation of ERK1/2, JNK, and p38 phosphorylation on cisplatin-induced rat-kidney injury with ameliorated oxidative stress, apoptosis, and inflammation. Moreover, daidzein modulated NF-κB, p38, MAPK and TGF-β1 pathway to counteract angiotensin II-induced inflammation on mice^29^. In line with previous findings, the present results showed that supplementation with daidzein inhibited the overexpression of *p38*, *JNK*, *ERK* and *NF-κB* induced by SBM, which suggested that daidzein might attenuate the inflammation via modulating MAPKs and NF-κB signaling.

To resist the assault of reactive oxygen species (ROS), an antioxidant system, including enzymatic and non-enzymatic antioxidant of fish have developed to either prevent or repair the oxidative damage^33,34^, which is tight regulated by Nrf2-keap1 signaling^35^. In the condition of oxidative stress, Nrf2 translocates to the nucleus by detaching from Keap1, and bounds to antioxidant response element to induce the expression of antioxidant genes such as superoxide dismutase (*SOD*), *NQO-1* and *HO-1*^35^. In the current study, the mRNA levels of *HO-1*, *NQO*, *prdx-6,* as well as signal molecule *Nrf2*, were significantly up-regulated by SBM treatment, suggesting the intestine of turbot underwent oxidative stress. However, the up-regulated expression of those genes was down-regulated by 40 mg/kg daidzein in the diet. The results were contrary to previous finding, which showed that 25 μM daidzein enhanced the expression of antioxidant enzyme such as *SOD3* and *CAT* via up-regulating Nrf2 in human cancer cell MCF7, HeLa and SK-OV-3, concomitant with improved oxidative stress condition^20^. Indeed, previous studies revealed that daidzein could elevate antioxidant enzymes activities to inhibit oxidative stress^36^. Besides, daidzein is also a direct free radical scavenger, and possesses high antioxidant power to scavenge free radical such as superoxide anion radical, hydroxyl radical and hydrogen peroxide^37^. Thus, a possible explanation for the seemingly opposite results may be that daidzein acts as a free radical scavenger, resulting in alleviative oxidative stress of turbot caused by SBM. Accordingly, the antioxidative effects and daidzein on fish and the mechanisms involved merits further study.

The enhanced intestinal epithelial cells turnover and apoptosis usually emerged with SBMIE^11,38^. Proteins of the B-cell lymphoma-2 (BCL-2) family composed of anti-apoptotic proteins (Bcl-2, BCL-_XL_, ect.) and pro-apoptotic (Bax, Bid, BAX, etc.) control the intrinsic apoptosis pathway^39^. Once apoptosis initiates, caspases, a family of endoproteases playing an essential role in apoptosis, will be activated; caspases-8 and -9, initiator caspases, are activated by upstream signaling, which subsequently activated executioner caspases-3, -6 and -7 to induce efficient cell death^40^. In the current study, the up-regulated gene expression of *Bax*, *Bid*, and *caspase-9* in SBM group was significantly inhibited by dietary daidzein, indicating a suppression of apoptosis. The results were in line with a previous study on 7,12-dimethylbenz[*a*]-anthracene (DMBA) treated mice, which showed that daidzein restored the increased Bax and caspase-3 caused by DMBA to normal values^41^. Aras et al.^42^ also provided evidence that administration of daidzein decreased caspase-3 and caspase-9 immunoreactivity in the brain of rat suffered middle cerebral artery occlusion. TJs protein are the apical-most adhesive junctional complexes in epithelial cells, which constitute a major competent of intestinal physical barrier together with intestinal epithelial cells^43,44^. In this study, the decreased expression of barrier-forming TJs *claudin-4*, *JAM-1* and *ZO-1* transcript variant 1 observed in SBM group were in accord with previous studies on turbot^3,4,13,14^, indicating a disrupted TJs barrier. As expected, dietary daidzein reversed the changes of those gene expression. The result was in agreement with Ou’s^27^ finding which showed that dietary daidzein (fish meal-based diet) enhanced the intestinal mucosal barrier function of turbot via significantly up-regulating *tricellulin*, *ZO-1*, *claudin-like* and *occludin* expression. These findings suggest that daidzein could inhibit apoptosis of epithelial cells and modulate TJs to maintain the intestinal structural integrity.

The intestinal bacterial community was also included in present study, similar with previous studies which showed that dietary daidzein could modulate intestinal bacterial community^27^. The results showed that dietary SBM significantly increased the abundance of Bacteroidetes, and decreased the abundance of Proteobacteria compared with FM group. The increased abundance of Bacteroidetes was consistent with previous studies carried out on turbot fed with soybean meal^3,10,14^. Bacteria of Bacteroidetes phylum possess a lot of genes encoding for carbohydrate-degrading enzymes, and are considered primary degraders of polysaccharides^45^. Bacteroidales S24-7, a family of Bacteroidetes, accounting for 53.00% of intestinal bacterial community in SBM group in present study, have been proved owing the ability to degrade complex carbohydrate^46^, which might help to explain the rich abundance of Bacteroidetes in SBM treatment. However, most carnivorous fish species are considered to be “glucose intolerant”^47,48^, the improved the usability of carbohydrate might increase the glucose load of fish, which eventually disturbed glucose homeostasis, even induced inflammation and oxidative stress^49^. While, administration of daidzein significantly reversed the situation with boosted Proteobacteria and Deinococcus–Thermus instead of Bacteroidetes compared with SBM group and made the microbial composition close to the FM group in terms of beta diversity analysis. Although the increased abundance of Proteobacteria is usually found to be associated with many human disease^50,51^, some marine Proteobacteria can produce bioactive compounds with anti-cancer and antibiotic activity^52^. Proteobacteria are the prominent gut microbiota in intestine of fish^53^, and its relative abundance is even up to 90.64% in turbot^54^. *Sphingomonas*, the most predominant genus in daidzein group, mainly contributed to the increment of Proteobacteria in the current study. Previously, *Sphingomonas* was found to be abundant in the intestine of healthy Atlantic salmon compared with unhealthy fish^55^. *Sphingomonas* is metabolically versatile, which can utilize many kinds of compounds and some environmental contaminants such as hydrophobic polycyclic aromatic hydrocarbons^56,57^, and was considered as a potential probiotic in aquaculture due to the removal of ammonia nitrogen and inhibition of *Vibrio* spp^58^. Bacteria from the phylum Deinococcus–Thermus are highly resistant to extreme stresses such as radiation, oxidation, desiccation and high temperature. Some studies revealed that Deinococcus–Thermus bacteria such as genus *Thermus*, can synthesize carotenoids^59–62^ which can reduce oxidative stress by interrupting the propagation of free radicals. The prevalence of them in daidzein treatment might contribute the antioxidant ability of daidzein. Moreover, the relative abundance of a lactic acid-producing bacteria *Lactococcus lactis*, was significant increased by dietary daidzein. The increased abundance of lactic acid bacteria was also observed in the intestine of turbot fed fishmeal-based diets containing 400 mg/kg daidzein^27^. As a probiotic, *Lactococcus lactis* is widely studied in aquaculture, Adel et al.^63^ found that diets containing *Lactococcus lactis* subsp. *Lactis* improved the growth performance, disease resistance, digestive enzyme activity and intestinal microbiota of white shrimps (*Litopenaeus vannamei*). Compared with SBM group, dietary daidzein significantly decreased the relative abundance of potential pathogenic bacterium *Alistipes* and *Lachnospiraceae* NK4A136 group. *Alistipes*, a genus of Bacteroidetes, one of the most abundant genera, existed in human colorectal adenoma^64^. Previous studies demonstrated that *Alistipes* could produce lipopolysaccharide (LPS)^65^ and promote inflammation and tumorigenesis^66^. A recent review done by Parker et al.^67^ indicated that *Alistipes* are highly related to dysbiosis and disease. The abundance of *Lachnospiraceae* NK4A136 group is positively correlated with gut dysbiosis^68^. In study of Cui et al.^69^, the abundance of *Lachnospiraceae* NK4A136 group was lower in ameliorated gut dysbiosis. However, dietary daidzein also down-regulated the abundance of *Bacteroides* and *Bacteroides acidifaciens*. By a meta-analysis, Zhou and Zhi^70^ revealed lower levels of *Bacteroides* in IBD patients. *Bacteroides acidifaciens* was beneficial for treating insulin resistance and preventing obesity in mice^71^, while its function on fish was unclear. All in all, these results suggested daidzein had a deep influence on intestinal microbiota of turbot, and the influence of daidzein on intestinal microbiota in fish merits further investigation.

In conclusion, the present study suggested that 40 mg/kg daidzein in the diet could remarkably mitigate the SBMIE of turbot. The dietary supplementation of daidzein displayed strong inhibition of key genes involved in inflammatory response, antioxidant enzyme, and apoptosis, improved intestinal integrity, and had a positive influence on intestinal microbiota profiles. Besides, daidzein might modulate p38, JNK and NF-κB signaling pathway to counteract SBM-induced inflammation. The present results indicate that daidzein is a promising feed additive for fish feed.

## Materials and methods

### Diets and fish husbandry

In this study, three isonitrogenous and isolipidic experimental diets containing 52.7% crude protein and 10.2% crude lipid were formulated (Table 2): fish meal diet (FM): containing 68% fish meal, SBM diet: with 40% fish meal protein in FM replaced by soybean meal, and DAID diet: 40 mg/kg daidzein added into SBM. All diets were stored at − 20 °C after drying for 12 h at 50 °C in a ventilated oven.

**Table 2:** Formulation and proximate composition of the experimental diets (% dry matter basis).

Ingredients	FM	SBM	DAID
Fish meal^a^	68	40.80	40.80
Soybean meal^b^	0	37.90	37.90
α-Starch	16	11.55	11.546
Fish oil	4.80	6.70	6.70
Soy lecithin	0.50	0.50	0.50
Vitamin premix^c^	1	1	1
Mineral premix^d^	0.50	0.50	0.50
Choline chloride	0.30	0.30	0.30
Monocalcium phosphate	0.50	0.50	0.50
Ethoxyquin	0.05	0.05	0.05
Yttrium oxide	0.10	0.10	0.10
Calcium propionate	0.10	0.10	0.10
Cellulose	8.15	0	0
Daidzein^e^	0	0	0.004
Sum	100	100	100
**Proximate composition**			
Dry matter content	97.07	97.11	97.75
Crude protein	50.41	50.15	51.46
Crude lipid	9.52	10.29	10.10
Content of daidzein (μg/g)	Nd	35.89	62.87

*FM* fish meal diet, *SBM* soybean meal diet, *DAID* 40 mg/kg daidzein included into SBM diet, *Nd* no detected.

^a,b^Purchased from Qingdao Seven Great Bio-tech Company Limited (Qingdao, China), Fish meal: crude protein, 74.04%; crude lipid, 9.97%; Soybean meal: crude protein, 53.12%; crude lipid: 2.12%.

^c^Vitamin mixture (mg kg^−1^): VA, 32; VB1, 25; VB2, 45; VB6, 20; VB12, 10; Niacinaminde, 200; Inositol, 800; Calcium pantothenate, 60; VH, 60; Folate, 20; VE, 240; VK, 10; VC phosphate, 2000; VD, 5; Antioxidant, 3; Microcrystalline cellulose, 6470.

^d^Mineral mixture (mg kg^−1^): Mg, 313; Fe, 79.10; Zn, 62.60; Mn, 46.60; I, 2; Se, 0.90; Cu, 6.40; Zeolite powder, 3485.

^e^Purchased from Shanghai Macklin Biochemical Technology Co., Ltd., the purity was more than 98.051%.

Healthy juvenile turbot was purchased from a local company in Weihai City (Shandong Province, China). After acclimating to the experimental conditions for 2 weeks, turbot with even size (9.55 ± 0.04 g), high vitality and normal appearance were randomly assigned to 9 tanks (300 L, 30 fish per tank). Fish were fed twice daily to apparent satiation (8:00 a.m. and 6:00 p.m.) for 12 weeks. During the feeding period, the water temperature was 19–25 °C, pH 7–8, salinity 23–26, dissolved oxygen > 7.0 mg/L, and NH_4_^+^–N < 0.3 mg/L. The protocols of animal care and treatment performed in this study has been approved by the Institutional Animal Care and Use Committee of Ocean University of China. Moreover, this study was carried out in compliance with the ARRIVE guidelines. All methods involved in present study were strictly conducted following the Guide for the Use of Experimental Animals of Ocean University of China.

### Sampling

Fish were anesthetized with eugenol (1:10,000) (purity 99%, Shanghai Reagent Corp, Shanghai, China) before sampling. Distal intestine for real-time quantitative polymerase chain reaction (qRT‐PCR) was collected from three fish per tank. The samples of intestinal microbiota were obtained under alcohol burner, and the surface of three fish per tank was sterilized by 75% alcohol. All samples were frozen in liquid nitrogen immediately, then stored at − 80 °C.

### qRT‐PCR

The RNA of intestine samples was extracted by using RNAiso Plus (9108; Takara Biotech., Dalian, China). Then, the quality of RNA was determined by electrophoresis and NanoDrop ND-1000 (Nano-Drop Technologies, Wilmington, DE, USA), evaluating the integrity and concentration, respectively. The RNA was reversed transcribed to cDNA with PrimeScript RT reagent Kit (Perfect Real Time, Takara, Japan).

The specific primers presented in present study were synthesized by TSINGKE (Beijing, China) and presented in Table 3. *β-actin* was used as reference gene. The program of the qRT-PCR was described in our previous papers^72^, with some modifications: 2 min at 95 °C, followed by 35 cycles for 10 s at 95 °C, 10 s at 58 °C, and 20 s at 72 °C. The qPCR assays were performed in a final volume of 20 μL containing 10 μL of 2 × SYBR Green Real-time PCR Master Mix [TB Green Premix Ex Taq II (Tli RNase H Plus)] (TaKaRa, Japan), 7.4 μL of dH_2_O, 0.8 μL of each primer (10 μM) and 1 μL of DNA template (100 ng/μL). The relative gene expression level was calculated via the 2^−ΔΔCT^ method^73^.

**Table 3:** Primers.

Target genes	Forward primer (5′–3′)	Reverse primer (5′–3′)	GenBank number
*TNF-α*	GGACAGGGCTGGTACAACAC	TTCAATTAGTGCCACGACAAAGAG	AJ276709.1
*TGF-β*	CTGCAGGACTGGCTCAAAGG	CATGGTCAGGATGTATGGTGGT	KU238187.1
*p38*	GAACGCCCCCAACATCTCTA	CTCGGCTGCTGTTATTCGCT	Zhao et al.^4^
*JNK*	CTGGTAGAGCAGGTAGGACA	CACAGAAGACACTGGAAGAA	Zhao et al.^4^
*ERK*	TCAACCACATACTGGGCATCC	TCGAGTCGGCCTTGAAGAA	Zhao et al.^4^
*NF-κB*	ACACTGCTGAGCTGAAGATC	CTCTGAGCCCATCAGGGTC	MF370855
*Nrf2*	CCACAACAACGCTCCCTTCACC	TTGGCCCTCTGCTCGTCTCTG	AWP13156.1
*HO-1*	ACGAGGGTCTGTCGTTCTTTGCC	CCTGGAGCGTCTTTACTGGTTTA	JX453446.1
*Prdx-6*	ACTGCCCGCTGTGTGTTTGTG	CGGCGTGGCAACCTTCTTCTG	ADJ57694.1
*NQO*	CCGTAAACCCACCCTGCCATATA	ACTGCAACGACTCCCTCTCCAAC	AWP04640.1
*GST-3-like*	GGGAGGTGAACTAAGCCTTGCG	CGGCGGGAATCTGCGTCATC	AWP14093.1
*SOD*	AAACAATCTGCCAAACCTCTG	CAGGAGAACAGTAAAGCATGG	MG253620.1
*CAT*	TATCTTCGTCCGCACTGTTG	AGAAACCCAGCCTCACTTTG	MG253621.1
*GSH-px*	CCCTGATGACTGACCCAAAG	GCACAAGGCTGAGGAGTTTC	AWP02885.1
*Bax*	AGCATCTTTGCTGACGGGAT	GCGCTCTCTGATGACCTGAA	MN782169
*Bid*	TCTGGTGCTCCTTTCCTT	CTTTGTCCGTCGGTTTCT	AWP08813.1
*Bcl-2*	TTCCTCAACTCTCAAAGCACAATTC	ATTACACTCGCTCGCCATTCC	MN782168
*p53*	TCAGTATTTTGAAGACCAGCACACA	GTCATCTCGGAGCCCAACTG	EU711045
*Caspase-3*	TTCTGCCATTGTCTCTGTGC	GCCCTGCAACATAAAGCAAC	JU391554.1
*Caspase-9*	CCCAGGACATGATCGACGAG	ACAATGGGAAGGCTCGACTG	KY979512.1
*Claudin-3*	GCCAGATGCAGTGTAAGGTC	CCGTCCAGGAGACAGGGAT	KU238180
*Claudin-4*	ATGTGGAGTGTGTCGGCTT	AGACCTTGCACTGCATCTG	MF370857
*Claudin-7*	CTCCATCCTGCAGCTCAACA	GGTGCACTTCATTCCCATGC	MF370858
*Occludin*	ACTGGCATTCTTCATCGC	GGTACAGATTCTGGCACATC	KU238182
*JAM-1*	CCAAGATGGACACCGGAACT	CCTCCGGTGTTTAGGTCACG	MT787206
*ZO-1 transcript variant 1*	GAGTTTTCAGCTTCCGTGTT	AGAGAACCTGTCACTGATAGATGC	KU238184.1
*β-action*	TCCCTTCTATCGTCGGTCGCCCC	TCTCCATGTCATCCCAGTTGGTC	AY008305.1

*ZO-1* zonula occludens-1, *TNF-α* tumor necrosis factor-α, *p38* p38 mitogen-activated protein kinase, *TGF-β* transforming growth factor-β, *JNK* c-Jun N-terminal kinase, *ERK* extracellular regulated kinase, *NF-κB* nuclear transcription factor-kappa B, *Nrf2* Nuclear factor E2 -related factor 2, *HO-1* heme oxygenase-1, *JAM-1* Junctional adhesion molecules-1, *Prdx-6* Peroxiredoxin-6, *NQO* NAD(P)H quinone oxidoreductase, *GST-3-like* glutathione-*S*-transferase-3-like, *SOD* superoxide dismutase, *CAT* catalase, *GSH-Px* glutathione peroxidase.

### Bacterial DNA extraction, sequencing and bioinformatic analysis

Genomic DNA of intestinal bacteria was extracted using QIAamp DNA Stool Mini Kit (Qiagen, Germany) following the method described by Liu et al.^14^. The high-quality of DNA determined by integrity and quality via electrophoresis on 1.2% denatured agarose gel and NanoDrop ND-1000 (Nano-Drop Technologies, USA) was amplified with 515F (GTGCCAGCMGCCGCGGTAA) /806R (GGACTACHVGGGTWTCTAAT) primers for amplifying the V4 region of 16S rDNA of intestinal bacteria. The specific amplification program was presented in a previous work^14^. Sequencing was performed on an IlluminaHiSeq2500 platform and 250 bp paired-end reads were generated by Novogene Bioinformatics Technology Co., Ltd.

Paired-end reads were merged using FLASH (V1.2.7, http://ccb.jhu.edu/software/FLASH/) after cutting off the barcode and primer sequence. Then, high-quality clean tags were obtained via quality filtering performed under specific filtering conditions according to the QIIME (V1.7.0, http://qiime.org/index.html). After that the tags were compared with the Gold database (http://drive5.com/uchime/uchime_download.html) using UCHIME algorithm (http://www.drive5.com/usearch/manual/uchime_algo.html) to detect chimera sequences. Finally, the effective tags obtained for OTUs cluster based on a threshold 97% sequence similarity by Uparse software (Uparse v7.0.1001, http://drive5.com/uparse/)^74^. At the same time, the representative sequence of each OUT was produced. For each representative sequence, SSUrRNA database of SILVA (http://www.arb-silva.de/) were used to annotate taxonomic information (threshold value was set as 0.8–1) by Mothur method. α-diversity and β-diversity analysis were calculated using QIIME (v. 1.7.0) and displayed with R software (v. 2.15.3, https://www.r-project.org/)^75^. The statistical difference of α-diversity and β-diversity indices between groups were tested by Tukey’s test and Wilcox’s test. NMDS analysis, PCoA and UPGMA based on weighted unifrac distances were included to compared the community composition similarity of samples. NMDS was displayed by vegan package, and PCoA by WGCNA package, stats packages and ggplot2 package in R software (v. 2.15.3). To determine the changes in bacterial community populations caused by dietary daidzein, differentially abundant taxa between SBM and DAID groups were confirmed via MetaStat analysis^76^ and LEfSe analysis performed by LEfSe software (LEfSe 1.0)^77^.

### Statistical analysis

All data except microbiota data underwent the Bartlett test and the Shapiro–Wilk *W* goodness of fit test for homogeneity and normality of variance. Then, one-way analysis of variance (ANOVA) of the data was performed via SPSS 22.0. Tukey’s test was applied for comparing the means among treatments, and the data were presented as means ± standard error. *P* < 0.05 was regarded as statistically significant.

## Supplementary Information

Supplementary Information.

## Data Availability

Raw reads of 16s rDNA sequencing were deposited to the National Center for Biotechnology Information (NCBI)’s Sequence Read Archive (SRA) under Accession No. PRJNA645003.
