# Supplementary material for: The protective role of daidzein in intestinal health of turbot (Scophthalmus maximus L.) fed soybean meal-based diets
Source: Sci Rep. 2021 Feb 8;11:3352. doi: 10.1038/s41598-021-82866-1 (PMC7870896; doi:10.1038/s41598-021-82866-1)
Supplement: Supplementary file 1 — Supplementary Information. [file 41598_2021_82866_MOESM1_ESM.pdf]

Supplementary Materials

The protective role of daidzein in intestinal health of turbot (*Scophthalmus maximus* L.) fed soybean meal-based diets

Guijuan Yu[1], Yang Liu[1], Weihao Ou[1], Jihong Dai[1], Qinghui Ai[1], Wenbing Zhang[1], Kangsen Mai[1, 2] and Yanjiao Zhang[1, 2*]

[1] The Key Laboratory of Aquaculture Nutrition and Feed (Ministry of Agriculture), and the Key Laboratory of Mariculture (Ministry of Education), Ocean University of China, Qingdao, China,

[2] Laboratory for Marine Fisheries Science and Food Production Processes, Qingdao National Laboratory for Marine Science and Technology, Qingdao, China

*** Correspondence:**
Yanjiao Zhang
Tel./fax: +86 532 8203 1627;
E-mail address: yanjiaozhang@ouc.edu.cn (Y. Zhang).

Supplementary Figure S1.

Rarefaction curve of intestinal microbiota of juvenile turbot. FM, fish meal diet; SBM, soybean meal diet; DAID, 40 mg/kg daidzein included into SBM diet.

Supplementary Table S1

α-diversity index of intestinal microbiota of juvenile turbot. SBM, soybean meal diet; DAID, 40 mg/kg daidzein included into SBM diet. data were presented as means ± standard error, n = 3.

Supplementary Table S2

The relative abundance of *Bacteroidales S24-7* in group SBM and DAID (× 10^-2). Data were presented as means ± standard error, n = 3.

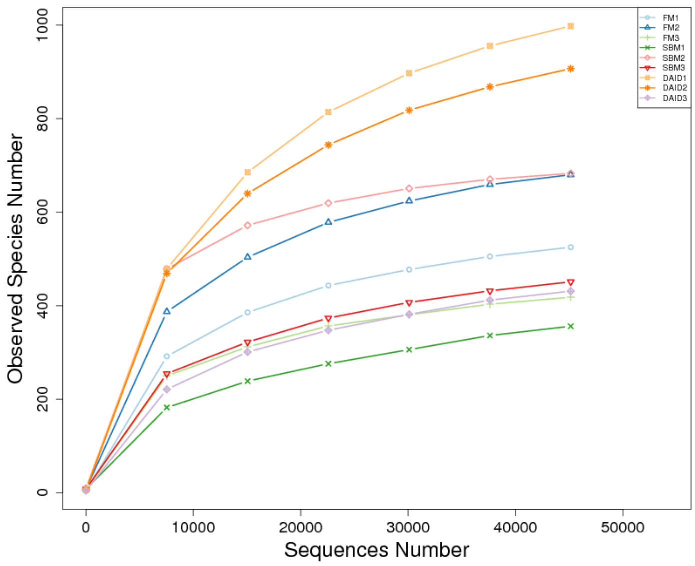

Supplementary Figure S1. Rarefaction curve of intestinal microbiota of juvenile turbot. FM, fish meal diet; SBM, soybean meal diet; DAID, 40 mg/kg daidzein included into SBM diet.

Sample name	Observed_species	Shannon	Simpson	Chao1	ACE	Goods_coverage
SBM	496.67 ± 97.12	5.83 ± 0.70	0.95 ± 0.02	573.59 ± 65.52	592.40 ± 60.85	1.00 ± 0.00
DAID	778.67 ± 175.81	4.12 ± 0.45	0.82 ± 0.05	855.77 ± 185.79	889.96 ± 191.94	1.00 ± 0.00

Supplementary Table S1 α-diversity index of intestinal microbiota of juvenile turbot. SBM, soybean meal diet; DAID, 40 mg/kg daidzein included into SBM diet. Data were presented as means ± standard error, n = 3.

Family	SBM	DAID
Bacteroidales S24-7	53.00 ± 11.15	0.25 ± 0.17

Supplementary Table S2 The relative abundance of *Bacteroidales S24-7* in group SBM and DAID (× 10^-2). Data were presented as means ± standard error, n = 3.
